# Dr. Gooch

**Published:** 1830-03

**Authors:** 


### OBITUARY.

We have to deplore the recent death of Dr. GOOCH, who was not less distinguished by his extensive acquirements as a general scholar, than his practical ability as a physician. The talent Dr. Gooch possessed as a writer was not so generally appreciated as it deserved to be, as his best and most finished compositions were published, anonymously of course, in the leading literary periodicals, and were known only to a few as the productions of his pen. Dr. Gooch's recent work, "On some of the most important Diseases of Females," of which we gave a copious analysis, is one of the best specimens of medical literature of the present day.

